# The Intervention of Prebiotics on Depression via the Gut–Brain Axis

**DOI:** 10.3390/molecules27123671

**Published:** 2022-06-07

**Authors:** Qinghui He, Congcong Si, Zhenjiao Sun, Yuhui Chen, Xin Zhang

**Affiliations:** 1 Amway (China) R&D Centre Co., Ltd., Guangzhou 510730, China; barneywind@163.com; 2 Ningbo Tech-inno Health Industry Co., Ltd., Ningbo 315211, China; sicongcong@taiyi-nb.com (C.S.); sunzhenjiao@taiyi-nb.com (Z.S.); chenyuhui@taiyi-nb.com (Y.C.); 3 Department of Food Science and Engineering, Ningbo University, Ningbo 315211, China

**Keywords:** intestinal microbiota, prebiotics, GBA, depression

## Abstract

The imbalance of intestinal microbiota can cause the accumulation of endotoxin in the main circulation system of the human body, which has a great impact on human health. Increased work and life pressure have led to a rise in the number of people falling into depression, which has also reduced their quality of life. The gut–brain axis (GBA) is closely related to the pathological basis of depression, and intestinal microbiota can improve depressive symptoms through GBA. Previous studies have proven that prebiotics can modulate intestinal microbiota and thus participate in human health regulation. We reviewed the regulatory mechanism of intestinal microbiota on depression through GBA, and discussed the effects of prebiotics, including plant polysaccharides and polyphenols on the regulation of intestinal microbiota, providing new clues for the prevention and treatment of depression.

## 1. Introduction

The intestinal microbiota is the most essential component of the human body. They live in the low-oxygen gut and have an independent metabolic mechanism, which is inseparable from the information exchange of movement, metabolism, and life activities of the host [1]. Recently, the communication between the gut and brain has received wide attention. The gut–brain axis (GBA) was proposed by Banks et al. in a study on the regulation of cholecystokinin in frog skin [2]. The brain and gut often establish various connections in the human body for proper functioning. GBA serves as a bridge linking to the gastrointestinal tract and central nervous system. To illuminate the link between gut and brain, researchers found that more than half of the patients with irritable bowel syndrome (IBS) had stress-related behaviors like depression or anxiety, which shows the intestinal microbiota participates in psychiatric symptoms [3,4,5]. The components of GBA include intestinal microorganisms and their metabolites, the intestinal tract, sympathetic and parasympathetic branches of the intestinal nervous system, autonomic nervous system, the neuroendocrine system, and the central nervous system. The intestinal nervous system (ENS) is the basis for the intestinal microbiota to participate in the regulation of neurological functions, emotions, and neurological diseases [6]. Intestinal microbiota can transfer the information through the ENS to the vagus nerve and then reach the brain, and it also modulates neurotransmitters such as norepinephrine, serotonin, and dopamine release. Short-chain fatty acids (SCFAs) are one of the most common metabolites released by microbiota. Free SCFAs in the intestine can cross the blood–brain barrier (BBB) through monocarboxylic acid transporter to promote the hippocampal neurogenesis and elongation in the brain, which will alleviate physiological symptoms [7,8]. According to a great number of animal and clinical data published in the last decade, the intestinal microbiota could modulate the central nervous system (CNS) to influence psychological, cognitive, behavioral, and neurological functions [9]. Specifically, previous research showed that dissonance and disorder among intestinal microbiota are associated with Alzheimer’s disease (AD), depression, autism, Parkinson’s disease, and other neuropsychological disorders [10].

Depression is a common psychiatric illness usually accompanied by vomiting, anorexia, constipation, diarrhea, nausea, and other gastrointestinal side effects [11,12]. The etiological factor of depression is complex, but possible factors of the intestinal microbiome to influence depression development have been explored in several studies [13]. The imbalance of intestinal microbiota imbalance can alter GBA judgment and processing, leading to the occurrence of anxiety and depressive behavior [14]. David et al. changed the behavior and intestinal microbiota of mice after administration of high sugar, high fat, and antibiotics [15]. The number of exploratory behaviors and *Clostridium* spp. was significantly increased, while the number of anxious behaviors and *Bacteroides* spp. was significantly decreased. These indicators were related to cognitive flexibility, suggesting that changes in intestinal microbiota could lead to changes in cognitive function [16]. Additionally, infection with *Campylobacter jejuni* can result in the production of c-fos proto-oncogene (c-FOS) proteins (the neuronal activation marker) in the central and autonomic nervous systems, inducing anxiety and depression behaviors [17]. There is an increasing number of researchers who believed that depression is comorbid with inflammation. Anti-inflammatory drugs—like COX-2 inhibitors—are effective in severe depression [18], they can alter neurotransmitter metabolism by reducing the availability of neurotransmitter precursors and activating the HPA axis. Abildgaard et al. [19] treated with a probiotic mixture—involving *Lactobacillus* and *Bifidobacterium* species—found that probiotic treatment markedly reduced depressive-like behavior independently of diet. Intriguingly, this result was correlated with a reduction in the level of pro-inflammatory factors TNF-α, and IL-6. Moreover, inflammatory cytokines could activate the hypothalamus–pituitary–adrenal (HPA) axis and neuroendocrine dysfunction [20]. All of this shows the mechanism of the intestinal microbiota in the pathogenesis of depression. Therefore, it can be considered that microbial regulation and control may be the theoretical basis and effective approach for prevention, intervention, and cure [21].

Polysaccharides are bioactive macromolecular substances composed of monosaccharides and small sugars linked by glycosidic bonds, which can be used to maintain tissue structure, provide metabolic energy, participate in biosynthesis reactions, and regulate intestinal microbiota. It is ubiquitous in plants, animals, and microorganisms in nature [22,23]. Among them, plant polysaccharides have been widely studied for their abundant sources with low toxicity and health benefit effects [24]. Specifically, dietary fiber, as a polysaccharide, cannot be decomposed by gastric acid in the stomach but can be used as a substrate for the metabolism of intestinal microbiota. After being absorbed by intestinal microbes, dietary fiber affects the design and function of intestinal microbiota [25]. For adults, dietary fiber of 25–35 g per day plays an important role in maintaining the gastrointestinal function, protecting health, and reducing the risk of cardiovascular diseases and cancer-preventing metabolic diseases [26]. Furthermore, dietary polysaccharides have significant effects on brain function through GBA [27,28]. Polyphenols are well-known organic chemicals in the diet, they are converted into primary and secondary metabolites by microbiota in the colon and then participate in various physiological activities such as the liver, intestine, and systemic circulation [29,30]. Intestinal microbiota can improve the bioavailability and concentration of phenols by converting polyphenols into small molecules, making them easier to be absorbed into the human circulation and metabolism [31]. In turn, polyphenols can increase the growth rate of beneficial bacteria—they can provide “food” for the beneficial gut microbiota—and inhibit the proliferation of pathogenic bacteria, thus maintaining the homeostasis of the intestinal microenvironment [32,33,34]. Tea polyphenols (TPs), for example, can combine with the phospholipid bilayer of the cell membrane of some harmful bacteria (such as *Escherichia coli*) to inhibit the formation of biofilm, and the function of pathogenic bacteria, thus interfering with its growth and reducing its toxicity [35,36]. Surprisingly, the host can bring TPs into the brain tissue by drinking tea. (−)-Epigallocatechin gallate (EGCG), the most abundant in catechins, has been found distributed in the brain in mice following direct oral administration of labeled EGCG [37]. In previous reports, TP is transformed by intestinal microbiota and the resulting metabolites have significant neuroprotective effects [38], and the neuroprotective effect of TPs has become a hotspot of human health research [39]. We summarized some bioactive components and their possible mechanism in brain functions (Table 1).

## 2. The Mechanisms of Intestinal Microbiota and GBA on Depression

With the development of probiotics research, the mechanism of probiotics maintaining health is becoming clearer. In recent years, the most notable part is preventing pathogens from adhering to the intestinal surface, maintenance of the epithelial barrier, and modulation and proper maturation of the immune system [14].

### 2.1. The Composition of Intestinal Microbiota and Its Effects on the Brain

From newborn babies to adults to the elderly, there are different microbial compositions in the intestine. It can be said that intestinal microbes run through the changes in everyone’s life course. Newborns are sterile in the womb, after leaving the womb, their intestinal microbiota is mostly from the mothers, breast milk, and exposure to the environment. *Proteobacteria* and *Actinobacteria* are relatively dominant bacteria in neonatal intestinal microbiota [44]. In adults, the gut microbiota makes up about 0.3 percent of human body weight, the same as the human brain, but it contains about 100 times more genes compared to the human genome [45,46,47]. Clinical studies have found that the diversity of intestinal microbiota in patients with depression is reduced, especially the abundance of *Coprococcus* and *Dialister* [48], which is significantly different from that of healthy people [49]. It provides evidence for the connection between depression symptoms and intestinal microbiota. Jiang et al. [50] have analyzed the abundance of microbiota in depressed and healthy individuals. Results showed that the abundance of *Firmicum* was higher in healthy people, while the abundance of *Bacteroides*, *Proteus,* and *Fusobacteria* was raised in depressed people at the phylum level. What is more, depressed patients have a higher abundance of *Enterobacteriaceae*, *Rikenellaceae*, *Porphyromonadaceae,* and *Acidaminococcaceae* compared to healthy ones. Additionally, the abundance of *Prevotella* in depressed patients increased but *Faecalibacterium* and *Ruminococci* decreased at the genus level [51]. However, Lin et al. [52] found that patients with depression had fewer *Bacteroidetes* and more *Firmicutes* such as *Clostridium XI*, and the *Klebsiella*, *Streptococcus,* and *Prevotella* positively correlated with the Hamilton Depression Scale (HAMD) scores, a measure of depression. Given the significantly changed abundance of *Akkermansia* for human health and disease states, recent studies revealed that *Akkermansia* spp. abundances may play a role in the pathogenesis of depression [53]. Its role in the intestinal barrier permeability and protection from intestinal inflammation has been established [54]. The primary butyric acid manufacturer, *Faecalibacterium prausnitzii*, exhibits a significant positive correlation with mood. A recent study found that *F.prausnitzii* (ATCC 27766) exhibits antidepressant-like effects and increases IL-10 levels while decreasing IL-6 levels [55]. Filipe et al. found the proportion of *Enterobacteriaceae* and echinococcus, inflammatory bacteria, increased significantly in depressed patients compared with healthy people, while the proportion of *Faecalibacterium*, anti-inflammatory bacteria decreased [56]. These findings suggested a close relationship between depression and gut microbiota, with a significant intestinal microecological imbalance in patients with depression [57].

Studies have shown that the human neuroendocrine and immune systems can regulate emotional expression. Disrupted intestinal microbiota will affect the structure and function of emotion-related brain areas by leading to inflammatory responses and changes in neurotransmitters, thus causing abnormal emotions in patients with mental diseases [58]. Anxiety and depression behaviors are often accompanied by a damaged intestinal barrier or intestinal microecological imbalance. Elevated levels of cytokines have been linked to depression, anxiety, and other psychiatric disorders [59,60]. The specific mechanism of depression with inflammation needs to be further studied, whether mental illness is caused by changes in intestinal microecology, or whether mental illness itself may cause the increase in some cytokines. In addition, intestinal microbes also can regulate the development, differentiation, and homeostasis of the CNS, and further influence cognitive function and neurotransmitter release. In the study of germ-free mice, the lack of a mature nervous system resulted in a decrease in ganglion number, increasing the proportion of neurons in the enteric muscle layer [61]. Similarly, a sterile environment increases the expression of synaptophysin and postsynaptic density-95, proteins that form synapses and release neurotransmitters, leading to the absence of neuron excitability and reduced medical conditions such as anxiety [62,63,64].

The pathways through which intestinal microbiota participates in CNS communication are as follows. Firstly, intestinal cells directly produce related neurotransmitters. Intestinal chromaffin cells are involved in the secretion of 5-hydroxytryptamine (5-HT), which can produce more than 90% of 5-HT in the intestinal tract and participate in the communication of the CNS. Secondly, the gut microbiome can also affect the body’s metabolism of neuroactive chemicals, affecting the number of substances that enter the brain. Thirdly, intestinal microbes can also affect the CNS through the HPA axis. It has been reported that the brain neuroendocrine system can be affected by HPA imbalance caused by intestinal microorganisms, leading to an increase in anxiety-like behavior [65].

### 2.2. The Bidirectional Regulation Mechanism of GBA

**Figure 1:**
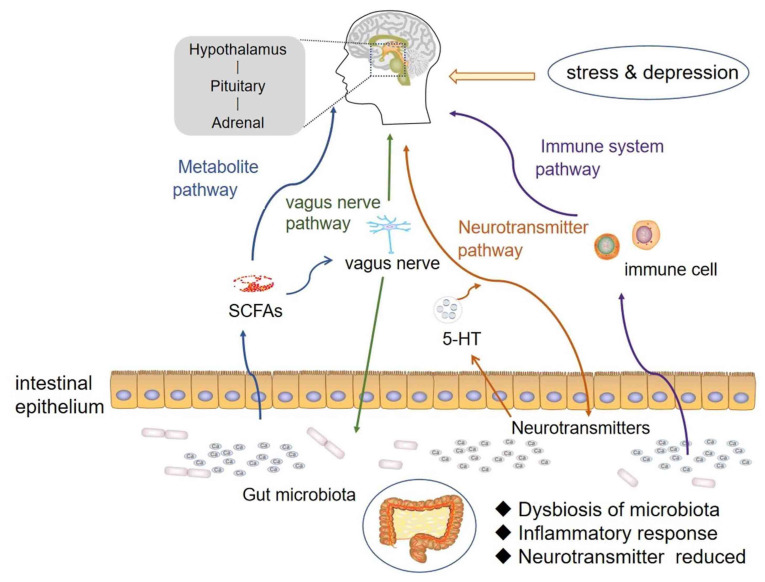
The bidirectional regulation mechanism of GBA.

Mainly, pathways of interaction between gut microbiota and the brain occur in the spinal cord. One is through direct communication between the autonomic nervous system (ANS) and the vagus nerve (VN) in the spinal cord, the other is to realize the indirect interaction between the gut and brain through the communication between the ENS [66]. The intestinal nervous system can form synaptic contact with the intestinal motor neurons through the sensory neurons and participate in the regulation of intestinal movement and secretion of intestinal hormones, which can also connect the intestine to the brain through synaptic release with the vagus nerve [67]. These feedback systems allow the brain to influence gastrointestinal function through movement, secretion, and mucin production. Overwhelming evidence corroborates that psychiatric disorders could contribute to the development and progression of chronic gastrointestinal diseases, environmental and stress also in the case of depression disease [62,68]. Mental stress can increase the frequency of cecal colonic rupture activity by interfering with the activity of intestinal microbiota. It can directly or indirectly alter the intestinal microbiota by affecting the release of neurotransmitters and hormones, mucosal permeability, and intestinal motility [69]. The neuroendocrine can transmit signals through the host intestinal microbiota to change the intestinal environment. The HPA axis plays a role in regulating the function of epithelial cells by guiding the brain, the mechanism of which is to influence intestinal permeability by secreting signaling molecules of neurons, immune cells, and intestinal chromaffin cells, thus changing the environment and composition of the intestinal microbiome. Then, the intestinal microbiota and its metabolites are involved in the regulation of behavioral activities controlled by the brain [13]. In the germ-free mice, *Lactobacillus* intervention significantly reduced the anxiety behavior and cognitive ability of the stressed mice, suggesting that the abnormal emotional behavior of the mice is related to the species richness of intestinal microecology [70]. Gut microbiota is involved in the synthesis of nutrients and proteins that regulate brain development [15,71]. Brain-derived neurotrophic factor (BDNF), a protein mainly expressed in the CNS can stimulate neurogenesis and regulate the formation and maintenance of synapses. It is also related to the influence of intestinal microbiota on host behavior through the HPA axis [72]. The intestine microbiota can differentiate neural signals including 5-HT, gamma-aminobutyric acid (GABA), and neuropeptides, which can play a key role in the regulation of the host nervous system [67,73]. These all suggested the bidirectional relationship between the brain and intestinal bacteria affects the body’s mood and activity (Figure 1) [74].

## 3. GBA on the Pathogenesis of Depression

### 3.1. Neurotransmitter Pathway

Intestinal microbiota can secrete GABA, catecholamine, histamine, and other neurotransmitters to transmit signals to the central nervous system through intestinal chromaffin cells and/or intestinal nerves [75,76], leading to behavioral and cognitive changes. Previous studies have shown that intestinal microbes can affect 5-HT neurotransmission in the CNS through humoral pathways [77]. In depressed people, the reuptake and metabolism of 5-HT are abnormal and the functions improperly [78]. 5-HT is not only involved in regulating myelin and synaptic formation, axon growth, and neuronal differentiation but is also essential in the onset and progression of depression. Intestinal microbiota could change tryptophan metabolism and affect the availability of peripheral tryptophan and the level of central tryptophan, which leads to changes in CNS 5-HT metabolism and influences the development of depression [79,80]. Animal studies exhibited the use of GABA_B_ receptors is linked to depressive-like behavior, it also sways the release of 5-HT [81]. *L. rhamnosus* have been proved to alter the central mRNA expression of GABAA and GABAB receptors via the vagus nerve [82].

### 3.2. Metabolite Pathway

SCFAs are critical metabolites from mice and human intestinal, in which acetic acid and butyric acid can affect the brain mood by stimulating bowel chromaffin cells to produce 5-HT and increase TPH1 expression [83]. In addition, SCFAs can promote the production and development of brain microglia of the CNS, maintain cell homeostasis and enhance the immune defense function of the brain [84]. Evidence showed that SCFAs can stimulate sympathetic nerves and ANS to interact with nerve cells through G protein coupling (GPR) receptor 41 (GPR41) and GPR43 [85]. Recent studies raised the question of vitamin B in the light of the symptoms of depression. Intestinal bacteria are critical sources of vitamin B involving niacin (B-3), biotin (B-7), and folate (B-9) [86]. B-3 and B-7 participate in systemic inflammation, while the B-9 levels in depression patients are lower than in controls. *Lactobacillus* and *Bifidobacterium* and other intestinal bacteria are major in synthesizing B-group vitamins [86]. Fatty acids may affect biological stress, and inflammation and cell structure play a role in depression. The levels of major fatty acids were significantly decreased in the serum of depression patients [87].

### 3.3. The Vagus Nerve Pathway

The vagus nerve pathway connects the abdominal cavity to the brain and can serve as a bridge between the intestinal microbiota and the central nervous system [88]. Interestingly, the low-grade colitis mouse model was activated by *Bifidobacteria longum* treatment, and the anxiety behavior and mood of the mice were improved, while this condition is not relieved in mice with a severed vagus nerve [89,90]. Similarly, the intact vagus pathway leads to down-regulation of C-FOS expression in cells after oral administration of *Campylobacter jejuni*, which also demonstrates that intestinal microbiota can alter host mood and behavior through the use of vagus afferent neurons. Perez-Burgos et al. found that long-term administration of *Lactobacillus rhamnosus* and stimulation of the vagus nerve reduced stress-induced cortisol levels [91]. The integrity of the vagus nerve underlies changes in the GABA system in the brain, and this exploratory behavioral increase is limited to specific shifts in the GABA system in the brain [92]. The cholinergic anti-inflammatory pathways are further triggered by depression-induced peripheral cytokine levels via vagal and basal forebrain neurons. In depressed patients, a hyperactive cholinergic system is linked to increased systemic inflammation [93]. *Lactobacillus rhamnosus* JB-1 supplementation can regulate aberrant total choline levels and relieve depressive-like behavior in rats [94]. Although there is considerable evidence that the vagus nerve acts as a signaling pathway from the microbiome living in the gut to the human brain, further specific mechanisms need to be studied and revealed on these pathways.

### 3.4. Immune System Pathway

There are many mechanisms of depression, among which immune dysfunction is associated with chronic inflammation caused by intestinal pathogens [93]. The research on the latter has confirmed that intestinal pathogens release some adverse metabolites, such as lipopolysaccharide, lipoprotein, and flagellin, which activate immune cells and promote the release of inflammatory mediators [94]. It has been shown that transgenic mice experience cognitive and anxiety-related behavioral changes during a behavioral task because they lack the lymphocyte recombination activator gene 1 (Rag1), but it is normalized by the combination of *Lactobacillus rhamnosus* and *Lactobacillus helveticus* [95]. In addition, gram-negative bacteria can bind Toll-like receptors (TLR-4) expressed on monocyte macrophages and microglia through their lipopolysaccharide (LPS) components to stimulate the production of pro-inflammatory cytokines such as IL-6 and IL-1B. Reduced intestinal permeability causes some of the bacteria that should be in the intestine to be transferred into the systemic circulation, where they stimulate TLR-4 on circulating immune cells, leading to an inflammatory response [96]. This is widely believed in cases of irritable bowel syndrome and depression [97]. Microglia, a characteristic immune cell that resides in the brain, can sense environmental changes in the brain through the vagal pathway and participates in regulation through neuroinflammation [98]. A recent study showed that the increase in microglia led to defect maturation and activation, thus ultimately to impaired immunity to bacterial or viral infections [99]. Therefore, maintaining the health of the intestinal may potentially mitigate adaptive damage to the immune system and behavioral changes. These findings suggest that keeping the gastrointestinal microbiome healthy and stable is essential for the proper functioning of the brain and healthy microglial metabolism for our physical health [100].

## 4. Prebiotics: Improve the Imbalance of Intestinal Microbiota to Ameliorate Depression

### 4.1. The Regulation of Depression by Plant Polysaccharides through Intestinal Microbiota

**Figure 2:**
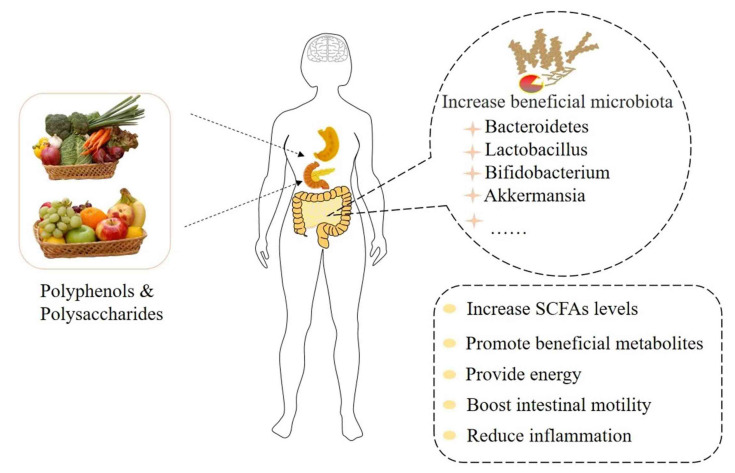
Prebiotics are converted by intestinal enzymes.

Some components of polysaccharides cannot be directly digested and absorbed by the human body due to the lack of polysaccharide degradation enzymes; however, the intestinal tract can degrade polysaccharides to make them easier to be absorbed and utilized by individuals (Figure 2). Polysaccharides can improve the composition and metabolism of intestinal microbiota, balance intestinal microecology, and boost the secretion of mucus by intestinal epithelium goblet cells to protect the gut, which is closely linked to protein expression and repair of the damaged intestinal barrier. The studies concluded that Ganoderma polysaccharides can increase the levels of IL-2 and IL-4 cytokines, which are indicators of immune status at the cellular level, and the intestinal role in immunity is improved by decreasing the level of serum diamine oxidase [101]. Several studies’ results suggest that plant polysaccharides as a new possibility for mental illness prevention and treatment strategies [102]. In vitro studies found that Lycium barbarum polysaccharide altered the composition of intestinal microbiota and significantly promoted the production of SCFAs during fermentation when simulating intestinal conditions. Among them, the relative abundances of *Bacteroides*, *Bifidobacterium*, *Phascolarctobacterium*, *Clostridium XlVb*, and *Prevotella* increased significantly [102]. Anaerobic fermentation of polysaccharides from flower tea significantly reduced the relative abundance of *Prevosiella* and *Clostridium XlVa*, and increased the relative abundance of *Klebsiella* and *Dialister*, thus significantly changing the microbial composition in the feces of healthy people [103]. Moreover, the purified components isolated from the raw polysaccharides of Fuzia tea could significantly increase the relative abundance of *Prevotella*, *Bacteroides*, and *Megasphaera* [104]. In addition, the regulatory effects of plant polysaccharides on the composition of intestinal microbiota have also been demonstrated in vivo. A team of researchers who analyzed the feces of mice found that a high-fat diet supplemented with fucoidan increased the number of beneficial bacteria in the gut of mice [105]. Ginkgo biloba leaves polysaccharides have been shown to reverse intestinal disorders associated with depression by increasing the richness of *Lactobacillus* species and may be used as a means of alleviating depression [106]. Okra polysaccharides can improve the intestinal microbiota turbulence of mice, reduce anxiety and behavior, and also intervene to increase the abundance of *Lactobacillus*, *Pasteurella*, and *Bacteroidetes* in mice intestinal [43].

The anti-depression effects of polysaccharides have been studied by many researchers. In the elevated plus-maze test, Liu et al. found that Lonicerae japonicae polysaccharides significantly reduced the time in the open arms as compared to the control group, and there was a negative correlation between the open arm and depression [107], and the expression of NLRP3, IL-1β, and Caspase-1 in the hippocampus of depressed mice was inhibited significantly. Lentinan can affect the level of AMPA receptor p-GluR1 in the hippocampus of depressed mice, leading to the up-regulation of GluR1 expression, thus exerting an obvious anti-depression effect [108,109]. In addition, Okra polysaccharides exert potential antidepressant effects by inactivating inflammatory responses in the colon and hippocampus, down-regulating TLR4/NF-κB pathway, promoting MAPKs signal transduction, and balancing intestinal microbiota [43].

SCFAs can influence neuroinflammation and associated psychiatric symptoms by affecting microglial morphology and function, which will impact mood. In particular, acetic acid and butyric acid play a major antidepressant role [110,111]. Polysaccharides from foods usually contain insoluble plant fibers including pectin, oligosaccharides, and inulin [42]. A growing number of researchers are finding that inulin can help relieve symptoms in people with type 2 diabetes by promoting the growth of intestinal microbiota and the release of SCFAs [112]. Pectin cannot be broken down by digestive enzymes and is easily absorbed by the intestinal microbiota, it is beneficial to the adhesion of *Lactobacillus* strains to epithelial cells and strengthens the mucus layer, thereby limiting harmful substances from entering the underlying tissues and thus preventing the activation of inflammatory responses [113]. Purple sweet potato polysaccharides have recently been shown to alleviate colonic inflammation in mice with colitis by blocking pro-inflammatory cytokines and regulating intestinal microbiota including *Lactobacillus* and *Bacteroidetes* [114]. With the help of intestinal sustenance, resistant starch produces much more butyric acid per unit time than any other dietary fiber [115]. Moreover, it may be used as the substrate for microbial fermentation in the large intestine as probiotics, allowing bacteria to proliferate at a normal or faster growth while cooperating with other dietary fibers to provide a probiotic effect [116]. With the addition of resistant starch, the number of *Bifidobacterial* in the intestinal tract was detected, which was positively correlated with the content of resistant starch within a certain range [117]. The relationship between resistant starch and intestinal microbiota has been examined, and it has been revealed that long-term lack of resistant starch intake may lead to intestinal microbiota disorder and various metabolic diseases. Dietary fiber, as the preferred substrate of epithelial cells, can produce butyrate, which can be used to improve the intestinal barrier and play a positive role in intestinal epithelial cells by lowering the concentration of local oxygen-inducible factors in intestinal epithelium with the help of intestinal microbes. Dietary fiber binds directly to immune-regulating receptors called Toll-like receptors, when it is ingested, the immune defense system becomes more alert. However, the relevance of these results remains uncertain due to the absence of valid evidence in humans clinically or for specific behaviors, genes, and so on [118]. Microbial metabolites (such as *Lactobacillus fermentum* NCIMB 5221) can release ferulic acid can also be produced after dietary fiber, which could regulate the intestinal composition, and release 5-HT into the bloodstream to play a role in overall health [119,120,121]. So far, there are few studies on the alleviating mechanism of dietary fiber on depression, and more in-depth studies are needed.

### 4.2. The Regulation of Depression by Plant Polyphenols through Intestinal Microbiota

As one of the essential prebiotics, polyphenols can stimulate the growth of beneficial bacteria and better build the intestinal microecology of the host. Flavonoids are abundantly present in tea, and the tea catechins account for the dominant position. The main types of catechins include (−)-epicatechin (EC) (−)-epigallocatechin (EGC), (−)-epicatechin gallate (ECG), and EGCG. In addition, quercetin, kaempferol, myricetin, and other flavanols and their glycosides have been reported. Catechins, the highest bioavailability in the tea, can suppress the pathogens of bacteria such as *Clostridium difficile*, *Clostridium perfringens*, *suppurative Streptococcus* spp., and *Streptococcus pneumonia* [122]. Sun et al. confirmed that TPs may not only participate in the bacteria growth and metabolism, but also interfere with the metabolism of cell membrane transport, transport, and energy supply, lowering bacterial toxicity and promoting the metabolic activities of intestinal microbiota [123]. In general, the conversion from catechins to hydroxyphenyl c-valerolactone can be further digested by gut microbes into smaller phenolic acids. TP can be metabolized by intestinal microorganisms and play a prebiotic role in microbial regulation [40]. Bioinformatics analysis of the data obtained revealed that the TP group of mice possessed more intestinal microbiota diversity and the content of *Bifidobacterium* spp. and *Lactobacillus* was significantly high, while *C. perfringens* and other *Clostridium* spp. were significantly reduced [124]. These results suggest that tea polyphenols can increase the abundance and diversity of some intestinal microbiota, making them better serve human health. However, different sources of tea polyphenols have bactericidal effects differ. Through in vitro evaluation of the regulation effect of TPs of green tea, Oolong tea, and black tea on the human intestinal microbiota, it was found that TPs of green tea, Oolong tea, and black tea could increase the number of Bifidobacteria in the fermentation broth. Within a certain range, fermentation time was positively correlated with the number of *Bifidobacteria*, among which TPs of Oolong tea had the most significant effect.

Dietary polyphenols have been found to act as neuroprotectants that possess pharmacological effects on the CNS for relieving anxiety and mitigating depression [125]. Robust evidence corroborates a bidirectional link between sleep disturbance and depression, also circadian dysregulation is one of the critical pathogenetic of depression nowadays. The main mechanism of its effect is that TPs can regulate the circadian rhythm via reversing the abnormal expression of circadian clock genes Bmal1 to relieve depression, and also regulate the intestinal microbiota to increase the number of probiotics to relieve depression [126]. By modulating monoaminergic neurons in the neurological system, TPs and their metabolites have been shown to lessen the prevalence of depression. TPs can inhibit the HPA axis overactivity by inhibiting the activity of monoamine oxidase B and reducing the serum corticosterone and adrenocorticotrophic hormone levels in mice [127]. Moreover, EGCG can improve the depression symptoms in mice, and its mechanism may be related to reducing serum corticosterone levels, inhibiting the generation of malonaldehyde in the hippocampus, promoting the secretion of SOD and GSH-PX, and down-regulating the expressions of IDO, IL-6 mRNA and IL-1β mRNA [128]. Polyphenols have anti-anxiety properties to some extent, depending on the dose. The antidepressant impact of TPs is dependent on the dose, type, and mechanism used.

Quercetin, which is a water-soluble and lipophilic flavonol, can easily cross cell membranes. In recent years, some papers revealed that quercetin and its glycoside derivatives have had good anti-depression activities. Quercetin can prevent depression and anxiety-like symptoms in rats induced by the maternal separation model [129]. Psychological stress caused by predatory stimulation is one of the depression models. A mouse model of depression was established with predatory stimulation for 3 days to study the antidepressant effect of quercetin, and the results showed that 50 mg/kg IP quercetin could improve the depression-like symptoms of mice [130]. In addition, Mehta et al. [131] found that 30 mg/kg of quercetin significantly reduced the expression of insulin and its receptor in the hippocampus and enhanced the expression of GLUT-4, thereby improving behavioral disorders in CUS model mice by regulating the hippocampal insulin signaling pathway.

It has been reported that quercetin derivatives have the potential for anti-depression by improving melanin corticotropin and reducing cytokines [41]. Studies have shown that a small part of quercetin glycoside derivatives such as isoquercetin, hyperin, and rutin have antidepressant activity. Thus, transforming quercetin glycoside into aglycone form is one of the methods to improve its bioavailability. Pharmacological effects of aglycone metabolites of quercetin were also evaluated, and 3, 4-dihydroxybenzoic acid, and p-hydroxyphenyl acetic acid were found to reduce immobile time in mice [132]. Ultra-high liquid chromatography-tandem mass spectrometry (UPLC-MS) was used to identify flavonoids, such as hyperin, as the effective substance basis for their potential antidepressant effects in animal brain tissues [133]. In the mouse model of maternal separation stress, rutin 100 mg/kg significantly alleviation the depression symptoms, which may be related to the decreased expression of NMDA receptors in model mice [134]. Flatoside is also a quercetin glycoside derivative, also known as quercetin-3-α-L- arabinofuran glycoside. In vivo studies have shown that intake of 2.5 mg/kg can improve SPT, shorten THE immobile time of FST and TST in CUMS model mice, and significantly reduce the levels of IL-1β, IL-6, and TNF-α in the hippocampus and the apoptosis rate of hippocampal neurons, thereby generating antidepressant effects [135]. In conclusion, quercetin and its derivatives have significant antidepressant activity at the animal level. Additionally, plant polyphenols and their derivatives may reduce the risk of depression in several ways. Prebiotics are becoming more and more popular in the market as a new antidepressant drug (Table 2).

## 5. Conclusions

The imbalance of intestinal microbiota is one of the pathogeneses of depression through GBA. More efforts are needed to illustrate the prevention mechanism for depression by regulating intestinal microbiota. Although the current review did not find an ameliorative effect of prebiotics on depression, these findings should be regarded as preliminary, given the relatively small number of eligible studies included in the analyses. Additionally, the intervention of plant polysaccharides and polyphenols to improve intestinal microbiota disorder provides a new idea for the relief of depression. Nevertheless, the stability, bioavailability, and metabolic transformation of plant polysaccharides and polyphenols remain to be solved. In the future, synthesizing plant polysaccharide and polyphenol derivatives with excellent properties, high bioavailability, stable metabolism, and significant biological activity will provide a reliable basis for the development of antidepressant drugs.

## Figures and Tables

**Table 1:** Summary of the modulatory effects of prebiotics on microbiota and brain.

Active Substance	Microbiota Involved	Effects on Brain	Possible Mechanism
Tea polyphenols [40]	*Bifidobacterium*	protect nerve cells, restrain neuronal apoptosis, EGCG can reach brain parenchyma through BBB in vivo	increase intestinal barrier function, stimulate the immune system, inhibit ROS and NO contents in rat midbrain and striatum
Quercetin [41]	*Firmicutes*, *Bacteroidetes*	Improved melanin corticotropin and reduced cytokines	*Firmicutes*/*Bacteroidetes* ratio decreased
Pectin [42]	*Lactobacillus*	strengthen the mucus layer, prevent activation of inflammatory responses	stimulate diversity of microbiota, improving intestinal integrity and mucosal proliferation
Okra polysaccharide [43]	*Bacteroides*, *Lactobacillus*	promote goblet cells of the intestinal epithelium, increase the expression of tight junction proteins and repair the damaged intestinal barrier	regulate the imbalance of intestinal microbiota

**Table 2:** Commercially available antidepressant products and their antidepressant mechanism.

Name	Active Substances (Prebiotics)	Possible Mechanism	Related Products
*Hyperici Perforati* *Herba*	Hypericin	Promote the expression of 5-HT and NE in the brain of stress-depressed rats	Extract of St. John’s Wort Tablets
*Acanthopanax senticosus (Rupr.etMaxim.) SHarms*	Syringin	Similar in potency to fluoxetine hydrochloride, overall improvement of depression-related somatic and core symptoms	Sultamicillin Tosylate Capsules
*Bupleuri Radix*	Saikosaponin	Decrease the amount of DA and 5-HT in the prefrontal lobe	Anle tablet, Jieyu Anshen tablet
*Acorus tatarinowii*	9-aminoacridine, kaempferol and cycloatunol	promote the activity and expression of CREB protein and mRNA in the hippocampal region, and then reduced neuronal apoptosis	Puyu Capsule, Antiyu and tranquilizing granules
